# Bacterial Infection and Predictors of Mortality in Patients with Autoimmune Liver Disease-Associated Acute-On-Chronic Liver Failure

**DOI:** 10.1155/2018/5108781

**Published:** 2018-01-28

**Authors:** Xuan Zhang, Ping Chen, Hainv Gao, Shaorui Hao, Meifang Yang, Hong Zhao, Jianhua Hu, Weihang Ma, Lanjuan Li

**Affiliations:** ^1^State Key Laboratory for Diagnosis and Treatment of Infectious Diseases, Collaborative Innovation Center for Diagnosis and Treatment of Infectious Diseases, The First Affiliated Hospital, College of Medicine, Zhejiang University, Hangzhou 310003, China; ^2^Infectious disease department, Shulan (Hangzhou) Hospital, Hangzhou, China

## Abstract

**Objective:**

To date, few studies are available on autoimmune liver disease-associated acute-on-chronic liver failure (ACLF). The aim of this study is to investigate bacterial infection and predictors of mortality in these patients.

**Methods:**

We retrospectively studied patients with autoimmune liver disease from August 2012 to August 2017. Clinical data of the patients were retrieved for analysis.

**Results:**

There were 53 ACLF patients and 53 patients without ACLF in this study. The ACLF group had a higher prevalence of complications (*P* < 0.05). The 28-day and 90-day mortality rates were also obviously high in patients with ACLF (38.3% and 74.5%, resp.) (*P* < 0.05). No predictor was significantly associated with 28-day and 90-day transplant-free mortality. In 53ACLF patients, 40 (75.5%) patients showed bacterial infection. ACLF patients with bacterial infection showed high Child-Pugh score, MELD score, CLIF-SOFA score, 28-day mortality, and 90-day mortality (*P* > 0.05). Moreover, C-reactive protein (CRP) using 12.15 mg/L cut-off value proved to be more accurate than procalcitonin in identifying patients with infection.

**Conclusions:**

Autoimmune liver disease-associated ACLF showed more complications and high mortality. Bacterial infection patients displayed a more severe condition than those without infection. Elevated CRP is an accurate marker for diagnosing bacterial infection in autoimmune liver disease-associated ACLF patients.

## 1. Introduction

In recent years, acute-on-chronic liver failure (ACLF) as a specific clinical form of liver failure has attracted increasing attention. In fact, ACLF is considered a syndrome that occurs on the background of chronic liver disease, and previously diagnosed cirrhosis is not required, which is characterized by acute hepatic decompensation resulting in liver failure (jaundice and prolongation of the international normalized ratio [INR]) and one or more extrahepatic organ failures that are associated with increased mortality within a period of 28 days and up to three months from onset [1, 2]. Moreover, ACLF can rapidly progress, requiring an urgent need for assessment and referral for liver transplantation [3]. Therefore, recognition and intervention of the predictors of mortality in ACLF patients can prevent or reverse the process and improve the survival rate.

Several patients with ACLF have been recently reported [3–7]. Moreau et al. found that bacterial infection is the trigger of 33% ACLF and is the most commonly identifiable trigger of this syndrome [4]. Some studies considered hepatic encephalopathy, low-serum sodium, and high INR as predictors of poor outcome in ACLF patients [3, 5]. However, few studies about autoimmune liver disease-induced ACLF patients are available to date. Except for the known predictors, possible risk factors have also received less attention. Moreover, bacterial infection in the population is not largely known. The aim of this study is, therefore, to collect data about autoimmune liver disease-associated ACLF patients to investigate bacterial infection and predictors of mortality to reduce the mortality in this population.

## 2. Materials and Methods

### 2.1. Study Population and Data Collection

We retrospectively analyzed the data of all patients admitted at the infectious disease wards of the First Affiliated Hospital, College of Medicine, Zhejiang University, China, and diagnosed with autoimmune liver disease-associated ACLF from August 2012 to August 2017. Patients with autoimmune liver disease who satisfied the 13th Asia-Pacific Congress of Clinical Microbiology and Infection (APCCMI) Consensus Guidelines for diagnosis and treatment of liver failure [8], but not diagnostic criteria of the European Association for the Study of the Liver (EASL) [4], were as control group (i.e., no ACLF group). All of the patients in our study had oral care after they admitted to hospital. Patients showing the following were excluded: coinfections with other viruses, including hepatitis A virus, hepatitis B virus, hepatitis C virus, hepatitis D virus, hepatitis E virus, and human immunodeficiency virus; concomitant liver diseases, such as Wilson's disease; coexisting liver cancer or extrahepatic malignancy; usage of hepatotoxic drugs. We used the composite of death or liver transplantation as our endpoint.

The research protocol was reviewed and approved by the Ethics Committee of the First Affiliated Hospital of Zhejiang University. The need for consent was waived because the study was retrospective and data were analyzed anonymously.

The following data were collected from hospital information system and medical documents: age, gender, diabetes, coexisting other autoimmune diseases, steroid exposure, laboratory findings, symptoms, the presence of bacterial infection at admission or during hospitalization, and mortality at 28 and 90 days. The chronic liver failure-sequential organ failure assessment (CLIF-SOFA) score, Child-Pugh score, and model for end-stage liver disease (MELD) score were calculated from the collected data.

### 2.2. Definitions

Diagnostic criteria of primary biliary cholangitis (PBC), autoimmune hepatitis (AIH), and primary sclerosing cholangitis (PSC) were defined by American Association for the Study of Liver Diseases (AASLD) [9–11]. Diagnosis of AIH-PBC overlap syndrome referenced the standard which was proposed by Chazouillères et al. in 1998 [12].

Diagnostic criteria and grades of ACLF were defined according to EASL definition [4], as follows.

*ACLF Grade 1. *This group includes 3 subgroups: (1) patients with single kidney failure, (2) patients with single failure of the liver, coagulation, circulation, or respiration who had a serum creatinine level ranging from 1.5 to 1.9 mg/dL and/or mild to moderate hepatic encephalopathy, and (3) patients with single cerebral failure who had a serum creatinine level ranging from 1.5 and 1.9 mg/dL.

*ACLF Grade 2. *This group includes the patients with 2 organ failures.

*ACLF Grade 3. *This group includes the patients with 3 organ failures or more.

The patients as control group satisfied the following criteria specified by the 13th APCCMI Consensus Guidelines for diagnosis and treatment of liver failure [8] as follows.

Patients with chronic liver diseases have acute or subacute deterioration of liver function. ACLF usually exhibits the following symptoms: (a) fatigue with gastrointestinal tract symptoms; (b) rapidly deepening jaundice, with total bilirubin 10 times higher than the upper limit of normal or a daily increase ≥ 17.1 *µ*mol/L; (c) hemorrhagic tendency with INR ≥ 1.5 or prothrombin activity ≤ 40% and other causes which have been excluded; (d) progressive reduction in liver size; and (e) hepatic encephalopathy occurrence.

Bacterial infection in parts of the body was defined as follows [13]. Bacterial pneumonia was defined as the association of clinical and radiological signs of lung infection observed in chest radiographs. Spontaneous bacterial peritonitis was diagnosed when ascites culture was positive or polymorphonuclear count was no less than 250 cells/*μ*L in ascites, excluding other inflammations such as pancreatitis, peritoneal carcinosis, tuberculosis, and bloody ascites. Urinary tract infection was diagnosed using bacterial culture positive or urine leukocyte count >15 cells/high power field and >10^6^ bacteria/*μ*L. Fever and cellulitis associated with leukocytosis were used to diagnose skin and soft tissue infection. Septicemia was defined as clinical signs of infection and two consecutive blood cultures yielding the same organism, when a blood culture yielding an organism was considered as bacteremia. Patients considered for bacterial infections but without positive culture or evidence of organ involvement were considered as undetermined infection.

### 2.3. Statistical Analyses

Statistical analysis was performed using SPSS version 18.0 (SPSS, Chicago, IL, USA). Categorical variables were expressed in percentages and frequencies. Continuous variables were expressed as means and standard deviation. Continuous variables were analyzed with independent-sample *t*-test when they in line with the normal distribution otherwise Mann–Whitney *U* test was used. The chi-square test was performed to analyze categorical variables. The 90-day mortality prediction was carried out with univariate and multivariate logistic regression. *P* value < 0.05 was considered statistically significant.

## 3. Result

### 3.1. Characteristics of the Study Cohort

During the study period, 53 patients with autoimmune liver disease-associated ACLF who were admitted to the infectious disease ward of our hospital were included in this study. Fifty-three patients were included as control group. The clinical features and laboratory results of the ACLF patients and no ACLF patients are shown in Table 1. From these 53 ACLF patients, 30 patients (56.6%) showed PBC, 21 (39.6%) displayed AIH, 1 (1.9%) had PSC, and one (1.9%) patients had AIH-PBC overlap syndrome. The control group included 26 (49.1%) patients of PBC, 24 (45.3%) patients of AIH, and 3 (5.7%) cases of PSC. Compared to patients without ACLF, the ACLF group had a higher prevalence of complications (*P* < 0.05), such as hepatic encephalopathy (54.7% and 3.8%, *P* ≤ 0.001) and bacterial infection (75.5% and 50.9%, *P* = 0.005). The 28-day and 90-day mortality rates were also obviously high in patients with ACLF (*P* < 0.05).

### 3.2. Risk Factors Associated with 28-Day and 90-Day Mortality

In the study, 6 (11.3%) patients underwent liver transplantation. In the remaining 47 patients, 28-day mortality and 90-day mortality were 38.3% (18) and 74.5% (35), respectively. For the 28-day transplant-free mortality, in univariate analysis, gastrointestinal bleeding (*P* = 0.025), hepatic encephalopathy (*P* = 0.044), and CLIF-SOFA score (*P* = 0.034) were significant factors (Table 2). However, in multivariate analysis, there was no predictor associated with increased mortality. In addition, for the 90-day transplant-free mortality, in univariate analysis, hepatic encephalopathy (*P* = 0.004), leukocyte counts (*P* = 0.046), hemoglobin (*P* = 0.048), aspartate aminotransferase (AST) (*P* = 0.021), serum creatinine (*P* = 0.016), serum sodium (*P* = 0.017), INR (*P* = 0.019), MELD score (*P* = 0.004), Child-Pugh score (*P* = 0.011), and CLIF-SOFA score (*P* = 0.001) were significant factors (Table 3). In multivariate logistic regression analysis, no predictor was significantly associated with 90-day transplant-free mortality (Table 3).

### 3.3. Clinical Features of Bacterial Infection in Patients with Autoimmune Liver Disease-Associated ACLF

In our study, 40 (75.5%) patients had bacterial infection, and 14 (35.0%) patients were diagnosed with bacterial coinfection in the first 72 h of admission. The demographic and clinical characteristics of ACLF patients with and without bacterial infection are detailed in Table 1.

ACLF patients with bacterial infection showed high Child-Pugh score, MELD score, CLIF-SOFA score, 28- day mortality, and 90-day mortality. Meanwhile, no statistical significance was observed (*P* > 0.05). However, in laboratory findings, bacterial infection patients displayed high leukocyte counts and C-reactive protein (CRP) (*P* < 0.05). High serum levels of serum creatinine and gamma glutamyl transpeptidase (GGT), low levels of hemoglobin, and alanine transaminase (ALT) were also observed in bacterial infection patients (*P* < 0.05) (Table 1).

In 40 ACLF patients with bacterial infection, the most common site of bacterial infection was in the respiratory tract (17, 42.5%), followed by the peritoneum, bloodstream, biliary tract, urinary tract, intestinal tract, skin and soft tissue, and undetermined site with percentage values of 22.5% (9), 7.5% (3), 7.5% (3), 5.0% (2), 5.0% (2), 2.5% (1), and 7.5% (3), respectively. The bacteriological evidence of infection was 7 cases (17.5%). The pathogens that caused bacterial infections were* Escherichia coli *in three cases, two of which produced extended spectrum *β* lactamase (ESBL);* Klebsiella pneumoniae* in two cases;* Staphylococcus aureus* in two cases, one of which involved methicillin-resistant* S. aureus*; and* Enterococcus faecalis* in one case.

### 3.4. Comparison of CRP and PCT in ACLF Patients with Bacterial Infection

**Figure 1:**
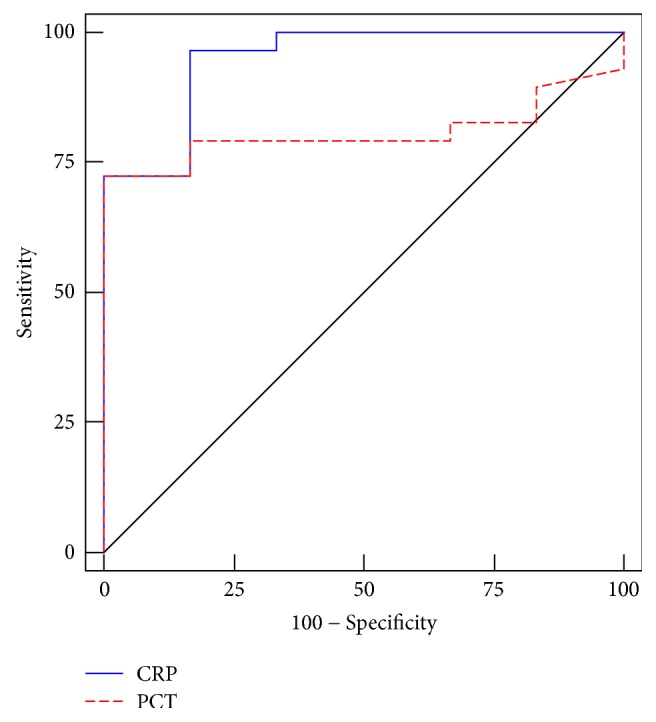
Using receiver operating characteristic analysis, comparison of C-reactive protein (CRP) and procalcitonin (PCT) in acute-on-chronic liver failure patients with bacterial infection.

CRP and PCT levels were obtained in 52 and 37 patients, respectively, among 53 patients. Thirty-seven patients had both biomarkers measured simultaneously. Using receiver operating characteristic analysis, area under curve (AUC) to diagnose bacterial infection was 0.948 (95% confidence interval [CI]: 0–1.000) for CRP (*P* = 0.001) compared with 0.807 (95% CI: 0.668–0.947) for PCT (*P* = 0.019) (Figure 1). For diagnosis of bacterial infection in CRP at a cut-off value of 12.15 mg/L, the sensitivity and specificity were 96.6% and 83.3%, respectively. For diagnosis of bacterial infection in PCT at a cut-off value of 0.57 ng/mL, the sensitivity and specificity were 72.4% and 100% respectively.

## 4. Discussion

ACLF is a hotspot issues; however, it is lack of uniform diagnostic criteria. Different diagnostic criteria could lead to different patient prognosis. In our study, ACLF patients satisfied the EASL definition, and patients who met APCCMI definition but not EASL definition were included as control group. The 28-day transplantation-free mortality of autoimmune liver disease-associated ACLF was 38.3%, and 90-day transplantation-free mortality was 74.5%. Of note, a great number of critically ill patients were included in the ACLF group; ACLF grades 2 and 3 (56.6% and 36.1%, resp.) were dominant. Moreover, similar to the CANONIC study [4], the ACLF group had a higher prevalence of complications and higher 28-day and 90-day mortality rates.

Several studies were available about predictors of mortality in ACLF patients [5, 14–19]. Yu et al. [14] found that age, etiology, serum sodium, and ascites are independently associated with mortality. Cárdenas et al. [5] considered that the presence of hyponatremia is an independent predictive factor of survival in patients with ACLF. In Mücke's study, infection-triggered ACLF was considered as an independent predictors associated with increased mortality [19]. In our study, no predictor was significantly associated with 28-day and 90-day transplant-free mortality. Small size of study population may cause the situation.

In this study, among the 53 patients with autoimmune liver disease-associated ACLF, 40 (75.5%) patients had bacterial infection. Bacterial infection in liver disease had been reported by previous documents, and the incidence was 24.4%–90% [13, 20–23]. However, study populations were different. Although bacterial infection in ACLF had been studied, autoimmune liver disease-associated ACLF received minimal attention.

In bacterial infection patients with ACLF, Child-Pugh score, MELD score, CLIF-SOFA score, 28-day mortality, and 90-day mortality were high in bacterial infection patients although no statistical significance was observed (*P* > 0.05). ACLF patients with bacterial infection showed a more severe condition than those without infection; this finding was consistent with that of previous study [13].

In recent years, serum CRP and PCT have been suggested for diagnosis and prediction of bacterial infection in chronic liver disease, with or without cirrhosis [13, 24–26]. Papp et al. [25] reported that CRP using a 10 mg/L cut-off value proved to be more accurate than PCT in identifying patients with infection (AUC: 0.93). In this study, we also revealed that CRP levels are more effective than PCT in the diagnosis of bacterial infection in autoimmune liver disease-associated ACLF. The optimal cut-off value of CRP for bacterial infection diagnosis is 12.15 mg/L (96.6% sensitivity and 83.3% specificity), with an AUC of 0.948, which is similar to previous report [25].

Several limitations were observed in this study. First, this work was a retrospective study at a single center. Some data collections were limited, and bacterial infection might be affected by specific circumstance of our institution. Second, some patients had been treated by antibacterial agents before admission, which might affect the accuracy of bacterial infection rate. Third, immunosuppressor except corticosteroid was not used in our study population. Thus, further studies are needed to investigate bacterial infection and predictors of mortality in autoimmune liver disease-associated ACLF patients using immunosuppressor.

## 5. Conclusions

In conclusion, autoimmune liver disease-associated ACLF displayed high mortality and had more complications. ACLF patients with bacterial infection showed a more severe condition than those without infection. CRP levels higher than 12.15 mg/L suggested bacterial infection in autoimmune liver disease-associated ACLF patients. No predictor was significantly associated with 28-day and 90-day transplant-free mortality. Further prospective and intervention studies on bacterial infection and predictors of mortality in autoimmune liver disease-associated ACLF patients with large sample numbers are thus needed.

## Figures and Tables

**Table 1:** Comparison of clinical features and laboratory results among the overall study collective.

Characteristics	No ACLF (*n* = 53)	ACLF (*n* = 53)	*P* value	ACLF		*P* value
				Infection group (*n* = 40)	Noninfection group (*n* = 13)	
Age	56.34 ± 11.64	58.23 ± 11.86	0.410	58.88 ± 13.18	56.23 ± 6.29	0.336
Female	46 (86.8%)	47 (88.7%)	0.767	34 (85.0%)	13 (100%)	0.317
Diabetes mellitus	9 (17.0%)	6 (11.3%)	0.403	5 (12.5%)	1 (7.7%)	0.635
Coexisting other autoimmune diseases	7 (13.2%)	7 (13.2%)	1.000	4 (10.0%)	3 (23.1%)	0.460
Steroid exposure	8 (15.1%)	11 (20.8%)	0.447	7 (17.5%)	4 (30.8%)	0.528
Complications						
Ascites	25 (47.2%)	41 (77.4%)	0.001	32 (80%)	9 (69.2%)	0.710
Gastrointestinal bleeding	3 (5.7%)	12 (14.0%)	0.026	8 (20%)	4 (30.8%)	0.671
Hepatic encephalopathy	2 (3.8%)	29 (54.7%)	≤0.001	21 (52.5%)	8 (61.5%)	0.570
Hepatorenal syndrome	2 (3.8%)	16 (30.2%)	0.001	15 (37.5%)	1 (7.7%)	0.092
Bacterial infection	27 (50.9%)	40 (75.5%)	0.005			
Laboratory data						
Leukocyte counts (×10 *∗* 9/L)	6.32 ± 3.93	10.43 ± 6.40	≤0.001	12.20 ± 6.34	4.97 ± 2.01	≤0.001
Hemoglobin (g/L)	99.06 ± 21.09	89.32 ± 19.51	0.015	86.30 ± 19.91	98.62 ± 15.39	0.047
Platelet (×10 *∗* 9/L)	108.83 ± 65.32	87.25 ± 52.30	0.063	89.70 ± 56.43	79.69 ± 37.68	0.472
CRP (mg/L)	19.25 ± 15.71	25.51 ± 19.05	0.071	30.49 ± 18.83	8.89 ± 5.93	≤0.001
PCT (ng/ml)	0.43 ± 0.44	1.25 ± 1.65	0.007	1.44 ± 1.76	0.33 ± 0.14	0.136
ALT (U/L)	227.06 ± 302.30	258.34 ± 331.79	0.613	177.15 ± 210.97	508.15 ± 492.74	0.034
AST (U/L)	288.42 ± 352.18	310.58 ± 290.45	0.724	270.48 ± 244.16	434.00 ± 387.24	0.172
AKP (U/L)	197.77 ± 105.19	1879.09 ± 104.54	0.361	176.05 ± 100.90	188.46 ± 118.91	0.714
GGT (U/L)	185.15 ± 199.38	143.72 ± 165.20	0.247	161.15 ± 183.58	90.08 ± 67.81	0.045
Total bilirubin (mg/dL)	20.44 ± 8.21	26.68 ± 8.20	0.005	26.16 ± 8.49	21.37 ± 6.21	0.067
Albumin (g/L)	27.53 ± 5.98	26.71 ± 3.30	0.372	26.59 ± 3.61	26.97 ± 2.22	0.723
Creatinine (mg/dL)	0.65 ± 0.19	1.22 ± 0.88	≤0.001	1.39 ± 0.91	0.72 ± 0.56	0.004
Fasting blood glucose (mmol/L)	4.68 ± 1.52	3.99 ± 1.99	0.119	3.80 ± 0.90	4.57 ± 3.74	0.471
Serum sodium (mmol/L)	136.60 ± 5.12	133.81 ± 5.59	0.009	133.03 ± 5.88	136.23 ± 3.79	0.072
INR	1.76 ± 0.29	3.04 ± 1.23	≤0.001	3.33 ± 1.54	2.95 ± 1.11	0.328
Child-Pugh score	9.53 ± 1.19	11.79 ± 1.46	≤0.001	11.06 ± 1.49	10.47 ± 1.85	0.055
MELD score	18.72 ± 3.15	29.60 ± 8.72	≤0.001	29.47 ± 7.06	27.37 ± 5.35	0.109
CLIF-SOFA score	7.43 ± 0.77	10.74 ± 2.22	≤0.001	11.03 ± 2.38	10.23 ± 1.54	0.266
ACLF grade						
Grade 1	42 (79.2%)	6 (11.3%)	0.186	4 (20.0%)	2 (15.4%)	0.678
Grade 2	0	30 (56.6%)	≤0.001	22 (55.0%)	8 (61.5%)	0.003
Grade 3	0	17 (32.1%)	≤0.001	14 (35.0%)	3 (23.1%)	0.008
28-day transplant-free mortality	4 (8.9%)	18 (38.3%)	0.001	15 (39.5%)	3 (33.3%)	0.733
90-day transplant-free mortality	6 (13.3%)	35 (74.5%)	≤0.001	27 (71.1%)	5 (55.6%)	0.618

CRP, C-reactive protein; PCT, procalcitonin; ALT, alanine transaminase; AST, aspartate aminotransferase; AKP, alkaline phosphatase; GGT, gamma glutamyl transpeptidase; INR, international normalized ratio; MELD, model for end-stage liver disease; CLIF-SOFA, chronic liver failure-sequential organ failure assessment.

**Table 2:** Significant univariate and multivariate logistic regression analyses of 28-day transplant-free mortality.

Variable	Univariate			Multivariate		
	OR	95% CI	*P* value	OR	95% CI	*P* value
Gastrointestinal bleeding	5.000	1.225–20.409	0.025	3.406	0.687–16.894	0.134
Hepatic encephalopathy	3.683	1.036–13.100	0.044	2.349	0.461–11.972	0.304
CLIF-SOFA score	1.362	1.023–1.812	0.034	1.093	0.741–1.613	0.653

CLIF-SOFA, chronic liver failure-sequential organ failure assessment.

**Table 3:** Significant univariate and multivariate logistic regression analyses of 90-day transplant-free mortality.

Variable	Univariate			Multivariate		
	OR	95% CI	*P* value	OR	95% CI	*P* value
Hepatic encephalopathy	8.800	2.024–38.253	0.004	36.714	0.085–15810.528	0.244
Leukocyte counts	1.160	1.003–1.342	0.046	1.328	0.962–1.832	0.085
Hemoglobin	0.964	0.930–1.000	0.048	0.912	0.808–1.029	0.135
AST	0.997	0.995–1.000	0.021	0.998	0.992–1.004	0.477
Creatinine	5.426	1.362–21.612	0.016	160.487	0.049–527959.376	0.219
INR	2.935	1.192–7.224	0.019	50.782	0.415–6206.850	0.109
MELD score	1.232	1.070–1.418	0.004	0.664	0.309–1.430	0.295
Child-Pugh score	2.003	1.169–3.430	0.011	0.595	0.114–2.457	0.473
CLIF-SOFA score	2.936	1.532–5.630	0.001	1.578	0.350–7.117	0.553

AST, aspartate aminotransferase; INR, international normalized ratio; MELD, model for end stage liver disease; CLIF-SOFA, chronic liver failure-sequential organ failure assessment.

## Abbreviations

ACLF: Acute-on-chronic liver failure
PBC: Primary biliary cholangitis
AIH: Autoimmune hepatitis
PSC: Primary sclerosing cholangitis
CLIF-SOFA: Chronic liver failure-sequential organ failure assessment
APPCCMI: Asia-Pacific Congress of Clinical Microbiology and Infection
EASL: European Association for the Study of the Liver
CRP: C-reactive protein
PCT: Procalcitonin
GGT: Gamma glutamyl transpeptidase
ALT: Alanine transaminase
INR: International normalized ratio
MELD: End-stage liver disease
AUC: Area under curve
CI: Confidence interval.
